# Spatial Distribution and Temporal Trend of Childhood Tuberculosis in Brazil

**DOI:** 10.3390/tropicalmed8010012

**Published:** 2022-12-25

**Authors:** Fernanda Bruzadelli Paulino da Costa, Antonio Carlos Vieira Ramos, Thaís Zamboni Berra, Yan Mathias Alves, Ruan Victor dos Santos Silva, Juliane de Almeida Crispim, Marcio Souza dos Santos, Adelia Roberto Nanque, Titilade Kehinde Ayandeyi Teibo, Ricardo Alexandre Arcêncio

**Affiliations:** Department of Maternal-Infant and Public Health Nursing, Ribeirão Preto College of Nursing, University of São Paulo, Ribeirão Preto 05403-000, SP, Brazil

**Keywords:** tuberculosis, infectious diseases, child health, epidemiology

## Abstract

Tuberculosis (TB) in children presents specificities in its diagnosis, which makes it prone to underreporting: therefore, the disease in this group is still a serious public health problem in several countries. We aimed to analyze the spatial distribution and temporal trend of childhood TB in Brazil. An ecological study with time series, spatial analysis, and description of cases in Brazil between 2010–2021 was conducted. A total of 1,054,263 TB cases were reported in the period, with 30,001 (2.8%) in children. The yearly average was 2,500 cases, with a trend toward an increase in the incidence rate in 2018 and 2019 and a decline in 2020. Children under 5 years old represented 38.2% of cases, 5.2% were indigenous, and 424 children (1.4%) died. Sputum culture was performed for 18.4% of pulmonary TB. The incidence rates were higher in municipalities in the north and midwest regions, with high occurrence locations (hot spots), especially on borders with other countries. There was a reduction in childhood TB in 2020, possibly related to the COVID-19 pandemic. Strategies are needed for the identification and monitoring of childhood TB, with reinforcement of professional training for assistance and control, especially in the most vulnerable locations and groups.

## 1. Introduction

Tuberculosis (TB) is an infectious disease caused by *Mycobacterium tuberculosis,* bacteria that predominantly affects the lungs and is transmitted primarily through the airways, especially by coughing [1]. It is currently one of the ten leading causes of death worldwide [1] and when considering children, the challenge to control the disease increases, mainly due to the perception that children are rarely are infected [2].

Since 2006, there has been an increase in the attention given to the specificities around TB in children and a greater recognition of its importance as a global public health challenge. Although most children are not responsible for widespread community transmission, the disease in this age group contributes to infant morbidity and mortality. In addition, children are possible future cases, and the disease in this group usually develops after one year of infection, which is a reason why its existence is an indicator of recent and continuous transmission in the community [3].

It is estimated that around 1 million children under the age of 15 will develop TB each year. As many non-specific symptoms overlap with other common childhood illnesses, many cases may not be easily identified. The main issue is the lack of access to diagnosis and the difficulty professionals face in detecting the disease among younger children. The challenge of diagnosing TB in this group is even more difficult due to the absence of a test that could be considered a gold standard, since bacteriological identification is not always possible. The literature shows that less than 20% of children with TB diagnosis have positive bacilloscopy and less than 50% of cases of *M. tuberculosis* are detected in culture [4].

The low positivity of bacteriological tests can be explained by the fact that the children are unable to voluntarily expectorate sputum, compromising the diagnosis in cases of pulmonary TB. The alternative method to obtain sputum in this situation is the collection of gastric lavage, a relatively invasive method. Considering the aforementioned challenges, it is common for the treatment of childhood TB to start based only on a triad that refers to the clinical and radiological imaging, the positivity of the tuberculin skin test, and contact with adults with bacilliferous TB [4].

Studies on TB in children suggest that the risks for infection are younger age, foreign-born parents, and prolonged close contact with the index case [5,6]. Severe disease can be particularly high in children co-infected with HIV and in those who have not received treatment [7,8], indicating the urgent need to strengthen interventions and better understand vulnerable areas and groups.

Among the Millennium Development Goals (MDGs), part of the 2030 agenda of the United Nations (UN) is the goal of ending the AIDS epidemic, TB, and other neglected diseases, and combating preventable deaths in children under 5 years of age, aiming to reduce child mortality [9]. Also noteworthy is the WHO strategy known as END TB, which seeks a world free of TB, with zero deaths, illnesses, and suffering due to the disease.

Despite the relevance of the topic, few studies developed to highlight the situation of TB among children and adolescents have been published [2,5,6,7,8], especially using tools such as spatial and temporal analysis, particularly with data from all over Brazil, which reveals an important gap in knowledge. 

This study is in line with the ongoing work on child and adolescent health surveillance in Brazil, which is in collaboration with the TB Research Network (REDE-TB), and aims to analyze the spatial distribution and temporal trend of childhood TB in Brazil.

## 2. Materials and Methods

### 2.1. Study Design

This is an ecological study with spatial and time series analysis and epidemiological description of cases.

### 2.2. Study Location

Brazil and its 5,570 municipalities. The country is the largest in South America and the fifth largest in the world in terms of area. Based on continental proportions, it extends over an area of 8,514,876.599 km² formed by the union of 26 states and a Federal District. It has a population of 212.7 million inhabitants according to the Brazilian Institute of Geography and Statistics (IBGE). 

The overall incidence of TB in Brazil was 32 cases per 100,000 inhabitants in 2021 [10] and the country is among the countries where the burden of the disease is considered high.

### 2.3. Study Population and Data Collection Sources

The study population are individuals younger than 15 years old, diagnosed with TB, and notified in the Brazilian Notifiable Diseases Information System (Sinan) from 1 January 2010 to 31 December 2021. Sinan is the national disease notification system in Brazil and contains data from the notification and investigation of cases of diseases and injuries that are included in the national list of compulsory notifiable diseases [11]. 

The data used are publicly accessible online and are available from the Ministry of Health (MoH) of Brazil via a public domain generic tabulator called TABNET/DATASUS [12], and were collected on 2 November 2022. The data downloaded were cleaned, processed, and analyzed.

For TB epidemiological surveillance in Brazil, only confirmed cases of the disease are notified, and these were the cases included in the study. A confirmed case is defined as one that, regardless of its clinical form, presents at least one positive sample in bacilloscopy, molecular test or culture (laboratory criterion), or any suspected case that did not meet the laboratory confirmation criteria, but presented results of imaging or histological tests suggestive of TB (clinical criterion) [13].

The surveillance recommendations for the disease also includes specifications for diagnosis of pulmonary TB in children and adolescents with negative bacilloscopy or a molecular test not detected, with an indication of evaluation of the clinical radiological image, contact with TB-positive adults, tuberculin test, and or poor nutritional status [13].

As for the treatment outcome of cases, the criteria for the cure, treatment abandonment, and death from TB were analyzed, following the definitions of the Brazil MoH [13].

For this study, individuals under 15 years of age were considered children, based on the age groups considered for data presentation by the World Health Organization (WHO).

### 2.4. Data Analysis

For the descriptive analysis of cases, categorical variables were included with calculations of absolute and relative frequency. Variables such as year of diagnosis, municipality, region of residence, age, sex, race, HIV testing, clinical form of disease, confirmation, treatment, and outcome were described.

TB incidence rates were calculated by month and year of diagnosis. For the municipalities of residence, we considered the numerator the number of TB cases in the particular period among children under 15 years of age, and in the denominator, the respective populations based on the projection of the IBGE [14] for the same period and age group, with a multiplication factor per 100,000 inhabitants.

For temporal analysis, time series of incidence rates were built according to the month of notification of each case, in Brazil and the regions, and then the seasonal decomposition of time series method was applied using LOESS (STL) [15].

The STL method decomposes a time series into three components: trend, seasonality, and noise. The trend refers to the direction in which the time series grows, according to a given time interval, and can follow a pattern of growth; decrease or stationarity. Seasonality is defined as identical patterns that a time series can follow and that are repeated regularly, at fixed periods. The “noise” represents the fluctuations observed during the period of the series, generally irregular and random, perceptible only when the other components of the time series are removed [16]. Of the time series components, we chose to select only the trend, with the aim to characterize the behavior of the TB incidence rate over time.

The TB spatial aggregation analysis was performed using the Getis-Ord Gi* technique, considering TB incidence rates in the group of children under 15 years of age according to their municipalities of residence. This technique indicates a local spatial association, taking the values for each municipality from a neighborhood matrix [17]. 

The interpretation of this statistic is based on the Z score (z-score) and the significance level values (α). A positive value of Z with statistical evidence indicates a spatial grouping of greater occurrence of the event (hot spot), while a negative value and statistical evidence of Z indicate a grouping of lesser occurrence of the event (cold spot). Confidence levels of 90%, 95%, and 99% were adopted.

Analyses were performed using the R Studio software (Posit, PBC / Boston, USA), forecast package, Epi Info^TM^ 7.2.4.0 (CDC USA) and Microsoft^®^ Excel^®^ 2016. For the distribution of rates, analysis of spatial aggregation and preparation of maps, the ArcGIS^®^ 10.8 software (Esri, California, USA) was used.

The study was approved by the Research Ethics Committee of the School of Nursing of Ribeirão Preto, University of São Paulo. By using aggregated secondary data available in public information systems, the study does not require the informed consent form (TCLE).

## 3. Results

From 2010 to 2021, 1,054,263 cases of TB were reported in Brazil, with 30,001 (2.8%) in children under 15 years of age. The annual average number of cases diagnosed in the period was 2500. The year with the highest number of diagnoses in children was 2019, with 2,854 cases, followed by 2011 and 2018, with 2,756 and 2,707 cases, respectively. In 2020, there was a drop in notifications, with 2,125 records.

There were more cases among males (52.2%), with a differentiation of the group of children aged 10 to 14 years, who were mostly female (54.4%). As for age, it should be noted that children under 5 years old accounted for 38.2% of cases and 40.9% were between 10 and 14 years old. The brown race represented most of the cases reported in the period (48.2%), but a notable proportion of cases in indigenous children (5.2%) stands out. As for the treatment outcome, the percentage of cure was 72.4% and 424 children (1.4%) died from TB, according to Table 1.

Of the TB cases in children under 15 years of age, 75.4% (22,613) were cases of pulmonary TB, and, of these, only 4,157 (18.4%) underwent sputum culture; those with a positive result were 1,869 (44.9%) of the tested cases. The percentage of cases with tests performed was higher in recent years, reaching 27.5% in 2021. As for the diagnostic tests for HIV, these were performed in 19,004 (63.3%) of the cases, of which 1,136 (5.9 %) were positive.

During the study period, it was recorded that 11,026 (36.8%) children underwent directly observed treatment (DOT), and this percentage was lower in recent years, reaching 27.0% in 2020.

**Figure 1:**
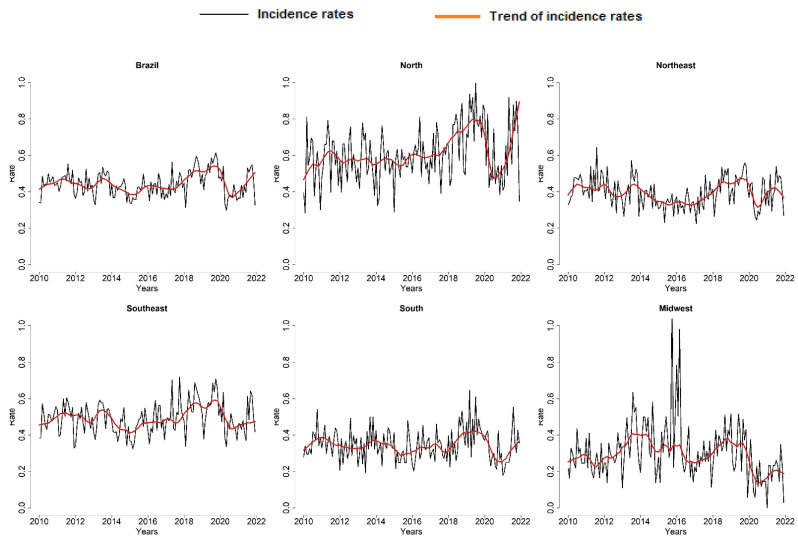
Time series and trend of TB cases in children in Brazil and regions (2010–2021). Source: TABNET/DATASUS/SINAN.

The monthly incidence rates of TB cases in children ranged from 0.29 to 0.61 cases per 100,000 inhabitants during the years studied, with higher rates being observed in the north and midwest regions of the country. Figure 1 presents the time series of cases and the trend for Brazil and its regions. It is possible to observe an increase in the disease in 2018 and 2019 and a trend toward a decrease in cases in 2020, a behavior similarly observed in all regions of the country.

**Figure 2:**
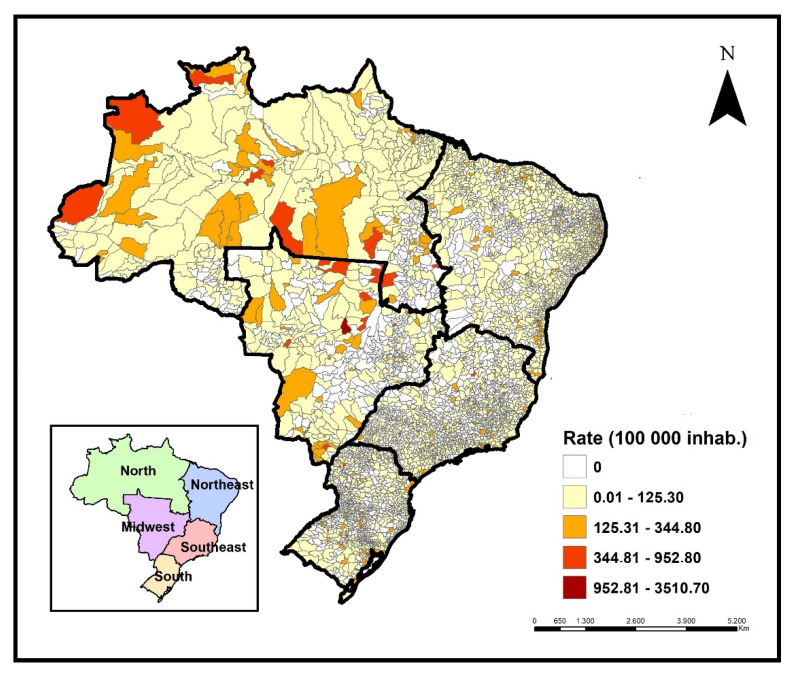
Distribution of TB incidence rates in children under 15 years of age according to the municipality of residence, Brazil, 2010–2021. Source: TABNET/DATASUS/SINAN.

Cases of childhood TB were registered in 2,839 Brazilian municipalities during the period, representing 51% of the country's territory. The states of São Paulo, Rio de Janeiro, Pernambuco, and Amazonas notified 51.2% of cases in children in the period, with 6164 (20.5%), 4,932 (16.4%), 2,215 (7.4%), and 2,069 (6.9%) cases, respectively. The highest incidence rates were registered in Amazonas, Roraima (north region), and Mato Grosso (midwest region). High incidence rates were identified in municipalities from these same regions (Figure 2).

**Figure 3:**
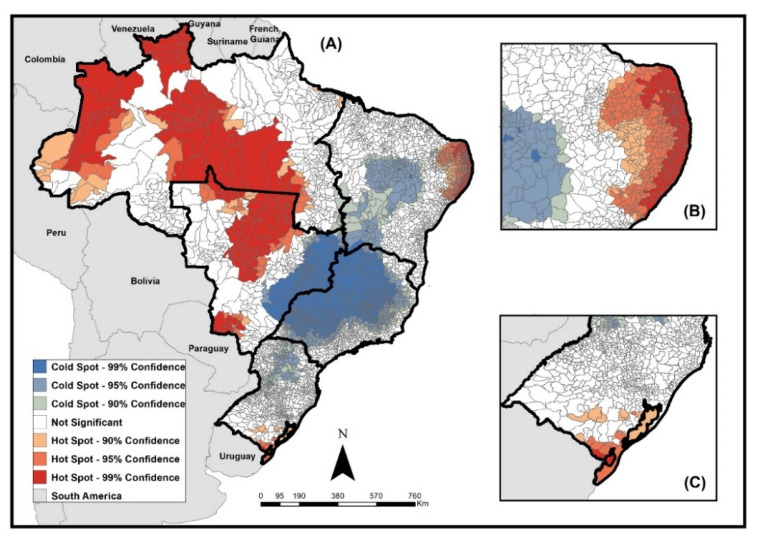
Clusters of high and low values of TB incidence rates in children under 15 years of age, according to the municipality of residence, Brazil, 2010–2021. Source: TABNET/DATASUS/SINAN.

Figure 3 presents the results of the analysis of the local spatial association of rates using the Getis-Ord Gi* technique, which allowed the identification of areas of high occurrence (hot spots) and low occurrence (cold spots) according to the municipalities of residence.

Areas of the high occurrence of the disease in children under 15 years of age were concentrated in municipalities in the north region, especially in regions bordering countries such as Colombia and Venezuela, and in municipalities in the midwest, north–east, and southern regions of Brazil. Some Brazilian states such as Amazonas, Roraima, Mato Grosso, Mato Grosso do Sul, and the coast of the states of Rio Grande do Norte, Paraíba, Pernambuco, and Alagoas stood out for the number of municipalities, with high occurrence in their territories.

The areas of low occurrence mainly included municipalities in the southeast region, especially in the states of São Paulo and Minas Gerais, and in the north of the southern region of the country, located in the states of Paraná and Santa Catarina.

## 4. Discussion

The study aimed to highlight the profile of childhood TB in Brazil through an epidemiological study with data from the last 12 years. An increase in the disease has been observed among children, in the opposite direction concerning public policies that seek to achieve the MDG target, especially concerning the control of the TB epidemic and the reduction in infant mortality [9], this contradicts the global END TB strategy that proposes “to end the global epidemic of tuberculosis” by 2035.

At the same time, it should be noted that in 2020, there was a reduction in the number of cases, possibly related to the COVID-19 pandemic, a scenario already pointed out by the Pan American Health Organization (PAHO). In their study, it is estimated that the diagnosis of new cases of TB in the Americas dropped from 15% to 20% during the year 2020 compared to the previous year, due to the pandemic, causing a situation that jeopardizes progress toward disease control [18].

Despite the relevant number of confirmed and reported cases under 15 years of age in the country, the percentage of TB cases in children of the total of the cases is low, especially when considering the 10% presented by the WHO for the whole world [19]. This low percentage may be related to the difficulty in identifying the disease and the difficulty in laboratory confirmation of TB in children.

The underreporting of cases in children may contribute to the permanence of the disease in the population, thus, camouflaging the real numbers that correctly describe the magnitude of TB, resulting in difficulty in applying strategies aimed at screening and early diagnosis among family members and the community, which aim to improve disease prevention in this age group [20]. 

The percentage of children with pulmonary TB who underwent sputum culture has grown in recent years but has not reached 30% of cases. It can be assumed that this low percentage is related to the difficulty of performing the test in some age groups, especially in children under 5 years old, who are often unable to voluntarily expectorate sputum, which can compromise the diagnosis of cases [4]. Considering underreporting and the difficulty of diagnosis in children under 5 years of age, who are the most vulnerable, the situation is quite relevant and points to the need to seek to better understand TB infection in this group. 

A highly recommended strategy to avoid the most severe conditions in younger children is the reduction in delays in the initiation of BCG vaccinations, with an increase in vaccination coverage at birth, based on the evidence that the vaccine substantially reduces mortality from childhood TB [21]. The BCG vaccine schedule in Brazil consists of administering a single dose, as early as possible, preferably in the maternity ward, right after birth. Unfortunately, it is known that in many locations there is a delay in the application of the vaccine and the pandemic may have affected vaccination coverage in Brazil, with BCG vaccination coverage being 73.51% in 2021, a much lower rate compared to the previous years, when coverage reached, on average, close to 95% of the target audience for the vaccine [22].

We evidenced a large number of cases in teenagers of 10 to 14 years, when the infection is already similar to that in adults [2,23]. It is possible to associate the transmission in this age group with the phase of the beginning of autonomy and mobility, and the higher incidence of TB at this age may be related to changes in behavior that are very common in this life stage, where sleep schedule, irregular diet, exuberant physical activities, and emotional lability can encourage the impairment of immune resistance. In addition, the expansion of the universe of conviviality and leisure in clusters/crowds increases the possibility of exposure to the bacillus [23]. For this group, the Xpert MTB/RIF is indicated, as a priority, for the diagnosis of pulmonary TB, considering that the majority have bacilliferous TB and already have the ability to expectorate adequate sputum samples for the exam.

Most cases occurred in males, but in older children, more cases were diagnosed in females, and it is pointed out in studies that females are more engaged in seeking health services since adolescence [20,24]. It is important to highlight that this applies to adolescents, but not to younger children, where parents or guardians condition the visit to the health service.

The number of confirmed cases in the brown race is in line with the profile of the Brazilian population and with findings in other studies [10,25]. Thinking about groups of social vulnerability, in addition to the brown race population, the proportion of cases in indigenous children (5.2%) is worrying when compared to the percentage of cases of the same race over 15 years old (0.9%) [12]. A 2019 study reveals that indigenous children and adolescents are subject to a high incidence of the disease, with marked regional inequalities, because most of the time, these groups are unable to access treatment, and monitoring by professionals is ineffective, also resulting in unfavorable outcomes regarding abandonment and death [26].

Although mortality among children is lower when compared to adults [12], it is worrying that 1.4% of the children diagnosed died of a treatable disease. In addition, it is expected that this number will be even higher, since it is known that the outcome of cases in the national notification system (Sinan) is still not ideal [27,28], with the Brazilian mortality system (SIM) being the most used to count deaths from this disease in the country. 

The percentage of cure in children was higher than in those over 15 years of age [12], but it is still low for the 85% ideally expected for disease control programs [29]. Treatment abandonment in children is lower than in adults [12], and it is important to consider its relationship with family care, as children depend on their parents or caregivers for treatment follow-up.

HIV positivity was lower than that found in other studies in children [30,31]. It is noteworthy that many did not perform or register the screening test for co-infection. It is known that the risk of developing TB in people living with HIV is much greater than the risk in the general population. This fact makes HIV the main comorbidity in patients with TB, imposing the need for greater surveillance in this population. This makes it essential that every child diagnosed with TB be tested for HIV and that every child with HIV is routinely tested for TB.

As for treatment, it was possible to monitor during the study period that the percentage of DOT performance was decreasing, which may lead to possible increases in abandonment, due to the lack of follow-up and proximity to patients. The DOTS strategy is considered by WHO one of the main factors responsible for the control of major progress in global TB [32] and must not be weakened. The low percentage of DOT completion in 2020 can be explained by the COVID-19 pandemic, as many families and professionals were socially isolated, leading to the removal of health services and the difficulty of carrying out home visits during the period. 

Spatial analysis helps in demonstrating priority locations for investments in public policies. The identification of hot and cold spots can be a useful health surveillance tool, as it allows investment in improving care in places with a high concentration of cases (hot zones) and improving the identification of cases in areas with possible underreporting (cold zones).

As for the spatial distribution of TB cases in children in Brazil, the incidence rates demonstrate the high occurrence of the disease in the north and midwest regions, which have hot spots for cases in several municipalities, especially in border areas. Borders are recognized as areas of vulnerability of countries to the entry and dissemination of potential threats to public health. The International Health Regulations (IHR 2005) highlight the importance of establishing international cooperation that allows the strengthening of joint epidemiological surveillance actions between countries that share common borders [33], and a study demonstrated the need for improvement and institutionalization of epidemiological surveillance actions on Brazil's land borders [34].

The regions with the highest rates are also known for the presence of indigenous territories, which raises the need for further studies in these populations. It is known that the persistence and occurrence of TB are related to socioeconomic indicators and seem to be influenced by the level of spatial aggregation, but also by particular characteristics of geographic areas [35]. Factors such as poverty, crowding, population density, and race have already been discussed in a study on the incidence of the disease in Brazil, and highlight the complexity of TB socioeconomic determinants [36]. 

Limitations of the study include the use of secondary data, which are susceptible to low-quality records. It was only possible to describe the variables available on the public data access platform, which does not cover all the fields of the case notification form.

## 5. Conclusions

In general, we can conclude that data on TB cases in children in recent years in Brazil point to some important deficiencies in the care and follow-up of cases in this group. These deficiencies include a low percentage of sputum cultures, HIV tests, and DOT, as well as the existence of relevant numbers of cases of abandonment and death, which lower the percentage of cure, and place the country far from controlling the disease and far away from the elimination goals agreed worldwide.

The results of the study allow us to highlight the need to improve strategies for timely identification and follow-up of childhood TB, reinforcing professional training to assist cases and better registration in information systems, in order to plan control actions. Given the above, strategies need to be aimed at professional training and awareness about the disease in this group, with emphasis on the training of professionals working in primary health care, the first contact with the cases. Better articulation and communication between health services providers in the country and in the borders is also necessary, in an integrated way, centered on the family and community.

We recommend the strengthening of surveillance and assistance services, especially for vulnerable groups and locations, where access to health care may be ineffective.

Few studies have dealt with the topic of childhood TB at a national level in Brazil, so we believe that this study can be a starting point for the problem to be prioritized and studied with other approaches.

## Figures and Tables

**Table 1:** Characteristics of TB cases in children under 15 years of age in Brazil (2010–2021).

Variables	No	%
**Age range**		
Less than 1 year	4799	16.0
1 to 4 years	6675	22.2
5 to 9 years	6252	20.8
10 to 14 years	12,275	40.9
**Sex**		
Male	15,654	52.2
Female	14,347	47.8
**Race**		
White	8265	27.5
Black	3018	10.1
Yellow	181	0.6
Brown	14,451	48.2
Indigenous	1563	5.2
Other ^1^	2523	8.4
**Treatment outcome**		
Cure	21,711	72.4
Abandonment	1947	6.5
Death from tuberculosis	424	1.4
Other outcome ^1^	5919	19.7
**Total**	**27,709**	**100**

^1^ Included ignored/blank. Source: TABNET / DATASUS / SINAN.

## Data Availability

Data used in this study can be found at: https://datasus.saude.gov.br/informacoes-de-saude-tabnet/. The information presented in this study is available on request from the corresponding author.
